# Alcohol and Disability Sports: Insights from Swiss Coaches

**DOI:** 10.2147/SAR.S528608

**Published:** 2025-08-15

**Authors:** Nikolai Kiselev, Florence Epiney, Linus Hany, Mariana Imhof, Regina Anton, Simon Amsler, Thomas Hurni, Simona Vallan, Susanne Dedial, Michael P Schaub, Corina Salis Gross

**Affiliations:** 1 Swiss Research Institute for Public Health and Addiction (ISGF), University of Zürich, Zurich, Switzerland; 2 PluSport Schweiz, Umbrella Organization of Swiss Disabled Sports, Volketswil, Switzerland; 3 Department of Sport Sociology and Management, Institute of Sport Science, University of Bern, Bern, Switzerland; 4 Faculty of Psychology, University of Basel, Basel, Switzerland; 5 Wheelchair Sports Switzerland (Rollstuhlsport), Umbrella Organization of Swiss Wheelchair Sports, Nottwil, Switzerland; 6 Procap Reisen & Sport, Procap Switzerland, Olten, Switzerland

**Keywords:** alcohol consumption, disability sports, individuals with disabilities, substance use prevention, participatory research, coaches

## Abstract

**Purpose:**

Inclusion in sports fosters social integration and well-being for individuals with disabilities. However, sports environments also present risks related to alcohol consumption, particularly through social interactions and established drinking norms. While general prevention programs exist in Swiss sports, limited research exists and no specific strategies are tailored to the needs of disability sports settings. This study explores alcohol consumption and prevention measures in Swiss disability sports, focusing on the perspectives of sports coaches.

**Methods:**

A qualitative research design was employed using semi-structured interviews with 27 coaches from disability sports programs in Switzerland. The thematic analysis by means of NVivo examined four key dimensions: perceptions of alcohol consumption, knowledge of alcohol-related risks, intervention approaches, and prevention strategies. The sample included coaches from diverse sports, disability groups, linguistic regions, and educational backgrounds.

**Results:**

Coaches exhibited varying perspectives on alcohol consumption, with some considering it irrelevant in disability sports and others recognizing its role in social settings surrounding sports activities. While alcohol use was largely rejected during training, it was often normalized in post-sports socialization. Knowledge of alcohol-related risks, particularly concerning its interaction with medication, was inconsistent among coaches, and few had received formal guidance on managing alcohol-related issues. Additionally, prevention measures were seldom implemented, and coaches’ awareness of the (national) prevention programs was unexpectedly low.

**Conclusion:**

The findings highlight a need for targeted prevention strategies in disability sports, particularly concerning alcohol consumption and its health implications for athletes with disabilities. Strengthening educational initiatives for coaches, integrating substance use prevention into training curricula, and fostering structured intervention strategies could improve awareness and promote safer sports environments. Future research should explore the perspectives of athletes with disabilities and examine broader policy implications to enhance prevention efforts in inclusive sports.

## Introduction

In Switzerland, physical activity and sports programs for individuals with disabilities (IWD) are vital in promoting inclusion, health, and well-being.^1^ The country’s sports programs are primarily coordinated by three major organizations: PluSport, the national umbrella organization for disability sports; Procap, which provides sports activities partly alongside other services for this population; and Wheelchair Sports Switzerland, which focuses on programs tailored for wheelchair users. Together, these organizations provide sports opportunities for more than 20,000 members through an extensive network of almost 700 regular sports groups and over 150 annual sports camps. These programs, which cater to a wide range of abilities and preferences, cover more than 50 different sports, from basketball and swimming to climbing and skiing.^2^,^3^

Participation in these programs is voluntary and resembles the structure of traditional sports clubs, with weekly sessions typically lasting one to two hours. Additionally, sports camps provide intensive training sessions along with opportunities for socialization and connection among participants from different regions, typically spanning several consecutive days to two weeks. All programs are guided by at least one qualified coach and an assistant coach (Coach for Disabled Sports [Behindertensportleitungsperson]). Coaches play a pivotal role in sports settings, acting as trainers and mentors influencing participants due to their close connection to them.

However, the landscape of IWD in Switzerland extends far beyond the reach of these programs. It is estimated that 1.7 million people in the country live with disabilities, with almost 600,000 considered to have severe impairments.^4^ The last survey of the sports clubs showed that 19% of Swiss sports clubs indicate that IWD are among their members.^5^ These findings, combined with the efforts of the three organizations mentioned above, demonstrate that IWD engage in sports both within and beyond the institutionalized disability sports system.

The participation of individuals in both specialized sports programs for people with disabilities and regular sports clubs highlights the evolving societal dynamics in Switzerland in recent years. Since the ratification of the United Nations Convention on the Rights of Persons with Disabilities (CRPD) in Switzerland in 2014, there has been a stronger emphasis on promoting inclusion across all public and private life areas, including sports.^6^

Diverse initiatives at various governmental levels have supported and encouraged better inclusion in sports. These efforts aim to create equal opportunities in terms of access to sports programs and across all aspects of club life, from coach roles and coaching positions to administrative and volunteer activities.^1^,^7–13^

But while inclusion in sports fosters health and social integration, it also presents unique risks for substance consumption, particularly in mixed settings where individuals with and without disabilities come together.^14^ Social gatherings after sports, often a routine part of club activities, can unintentionally expose vulnerable populations to higher risks of substance use – particularly alcohol.^15–18^ Alcohol is particularly relevant in the context of sports, as it is often culturally normalized during post-sports social gatherings (eg, traditional team celebrations, drinks sponsorships by alcohol companies in sports settings, membership meeting dinners, “social drink after sports”). Some studies even reported higher alcohol consumption in the settings around sports compared to the regular population.^19^,^20^ For IWD - especially those previously overlooked in prevention efforts - such settings might amplify their likelihood of substance consumption due to limited awareness or peer influence. Moreover, substance consumption might be exacerbated as a form of maladaptive coping strategy in response to various stressors in everyday life, as a means of self-medication for chronic pain, or due to simultaneous substance use related to medications commonly prescribed for this target population.^14^ Existing gaps in prevention and education for caregivers, sports coaches, and families further augment this risk.^21^,^22^

Although research on this topic is limited, existing evidence suggests that IWD - despite differences in consumption patterns between disability groups - consume substances at rates comparable to individuals without disabilities, but, at the same time, IWD may experience more severe health consequences related to alcohol use. These include increased vulnerability to medication interactions, higher risk for falls or accidents due to mobility impairments, and more pronounced effects on mental health.^14^,^23–82^ These factors underscore the importance of tailored prevention strategies and justify a closer examination of substance use in disability-specific sports settings.

Given that there was no research on substance use among the target population in Switzerland and almost no tailored prevention strategies or guidelines that address the risks adequately, the present study aimed at understanding the situation regarding substance use - especially alcohol consumption - within the context of the Swiss disabled sports system, as perceived by coaches working with this population. This position provides unique insights into patterns of substance use and the social dynamics within sports environments. As trusted figures, they are also well-placed for identifying early signs of risky behavior and implementing preventative measures, making their input invaluable for understanding and addressing substance-related risks in this context.

## Materials and Methods

This research was conducted as part of two master’s thesis projects in psychology at the University of Zurich. It represents one of two studies investigating perceptions of alcohol consumption and related risks within voluntary sports programs for IWD in Switzerland. These studies mark the initial phase of a larger project aimed at developing a prevention tool specifically designed to address the unique needs and conditions of disabled sports. This publication does not encompass a parallel study on voluntary program participants.^81^

Given the absence of comparable studies or publications examining coaches’ views on this subject, a cross-sectional qualitative approach utilizing semi-structured interviews was employed.

### Aims

The present study explores various aspects of alcohol consumption and understanding of consumption-related risks among IWDs participating in the structures of the Swiss disability sports system from the point of view of the coaches. The following research topics and questions were formulated for this purpose:

1. How do coaches of disability sports programs perceive alcohol consumption in disability sports?
2. What strategies for dealing with alcohol and potentially its application are known to coaches?
3. What do coaches understand about alcohol and its dangers/risks?
4. What resources are available to coaches to prevent/control alcohol consumption?
  - What resources do coaches wish for to better prevent/control alcohol consumption?

### Participatory Research Approach

This study employed a participatory research approach, engaging an expert group (distinct from the interview sample) of eight experts with diverse impairments from disability sports programs and eight coaches from various sports groups for IWD. Both the experts and the research team collaborated actively in formulating the research questions, designing the interview framework, analyzing the data, and interpreting the findings. This approach ensured that a wide range of perspectives and experiences were incorporated throughout the research process.^83^,^84^

### Respondents

Recognizing that IWDs are a heterogeneous group, we aimed to reach adult coaches of both genders training three distinct subpopulations: individuals with intellectual disabilities, individuals with physical disabilities/wheelchair users, and individuals with psychiatric disorders, both from sports clubs as well as sports camps. Given the topic, coaches of kids’ groups and camps were excluded from participation. Also, we aimed to include coaches from the German-speaking part of Switzerland and the second largest language group - French-speaking. Based on prior research in the field and supported by expert literature, we targeted interviewing approximately 20 to 30 coaches of voluntary sports groups for IWD (further coaches).^85–92^ Furthermore, participation was limited to no more than two interviewees from the same club or camp. These criteria ensured the diverse involvement of coaches from various regions and sports contexts.

### Interviewers

The interviews were carried out by two trained interviewers: a psychology graduate student from the University of Zürich conducted the sessions with German-speaking respondents, while a PhD student in sports science with an MSc in psychology from the University of Bern was responsible for the French-speaking respondents. Both interviewers had previous experience in qualitative data collection and underwent additional training tailored to free-listing interviewing methods. Neither interviewer had any prior personal connection with the respondents.

### Interview Questions

The semi-structured interview guide was developed through a collaborative, participatory, and iterative process involving both the expert and research teams. The guide was first drafted in German and subsequently translated into French by a professional translator who is a native French speaker. A bilingual sports scientist fluent in French and German reviewed the back-translation, and any inconsistencies were identified, discussed, and resolved. The participatory development process and the involvement of practitioners ensured strong face and content validity of the semi-structured interview guide. Given the exploratory nature of the study, the guide itself was not subject to psychometric validation procedures. However, the AUDIT-C was included as a standardized, validated screening instrument to assess alcohol consumption patterns.^93^

The interview guide comprised six main sections. The first section focused on brief introductory questions, including demographic and professional information such as age, educational background, work experience, role in disability sports, and descriptions of respondents and activities led. The second section addressed the relevance of alcohol consumption in disability sports, exploring its presence during sports activities, events, and social gatherings among both participants and coaches. The third section focused on responses and intervention readiness, including how interviewees would react to alcohol consumption or intoxication during sports programs or related events, as well as their awareness of existing prevention programs or organizational regulations. The fourth section assessed the need for preventive measures and examined societal perceptions of alcohol consumption among people with disabilities, exploring its potential impact on social inclusion. The fifth section briefly evaluated knowledge about alcohol, including risks, legal regulations, interactions with medications, criteria for identifying alcohol problems, and familiarity with healthy drinking strategies and support services. The last section broadened the scope to briefly include other substances and behavioral addictions, addressing tobacco, cannabis, medications, illegal drugs, sugary products, and non-substance-related dependencies within the context of disability sports. Last question specifically addressed respondents’ alcohol consumption and included the AUDIT-C and was assessed separately of previous data.^93^

### Recruitment

At the end of April 2023, 113 sports clubs and 15 sports camps were contacted via Email by the respective coordinators at PluSport, Procap, and Wheelchair Sports, inviting them to participate in the study. The current number of available camps was limited due to seasonality. The Email introduced the study and asking coaches of the sports groups to participate in the interview on the study topic. Interested coaches were instructed to contact the interviewers directly to schedule an interview. The data collection phase occurred from May to July 2023.

### Procedure

The interviews were conducted individually via Zoom or MS Teams. Informed consent was obtained prior to the interviews. The main portion of the interview guide was audio-recorded, with recordings deleted following transcription and anonymization. Personal information and responses to the AUDIT-C questionnaire were collected separately using a paper-and-pencil method by the interviewer and analyzed independently. As a token of appreciation, respondents received a compensation of 30 CHF (approximately 30 USD).

### Analysis

The thematic analysis was conducted using the NVivo software program (Version 14) from QSR International. We used applied thematic analysis as described by Guest, MacQueen, and Namey,^87^ as this approach allows for a structured yet flexible coding process and is particularly suitable for applied research settings and heterogeneous respondents groups, such as individuals with disabilities. The coding began after transcribing all interviews and thoroughly familiarizing the research team with the content. A coding framework was initially developed by reviewing three selected interview transcripts. The codes emerged directly from the data and were refined iteratively through the addition of subcodes and necessary adjustments. The framework was reviewed and, where required, revised in collaborative discussions with the research and expert teams before being applied to the full dataset. Two external reviewers with expertise in NVivo, thematic analysis, and disability sports, who were not involved in the initial coding, independently evaluated the final codes. Any discrepancies identified were discussed within the team, and adjustments were made as needed. It is essential to note that it was possible to allocate one statement to multiple codes, as each statement could contain information related to several themes.

## Results

Out of the 113 clubs and 15 camps contacted, 27 coaches from 19 camps or clubs agreed to participate (13 female / 14 male). The median age was 42 years (*Mdn=*42), between 19 and 66 years old. The interviews lasted between 30 and 60 minutes. The sample comprised German-speaking (n=20) and French-speaking (n=7) individuals. Educational background was split almost equally among vocational education (n=10), bachelor or comparable (n=9), and master or comparable (n=8). Most respondents were affiliated with PluSport (n=18), followed by Procap (n=6) and Wheelchair Sports (n=3), and reported 7 years of experience as a coach of a specific group or camp on median. Most came from sports groups (n=14) with mixed subgroups of participants, with the majority of participants having an intellectual disability. Three interviewees were from groups for participants with an intellectual disability only. Another six were coaches from groups for individuals with physical disabilities, and finally, four coached groups of individuals with psychiatric disorders. Besides three persons who coached both camps and groups, there were 21 from sports groups and six from sports camps.

### Own Reported Substance Consumption

Four out of 27 of the respondents reported not consuming alcohol. A closer examination using the AUDIT-C questionnaire revealed that half of the respondents (n=14) exhibited risky alcohol consumption. Next to alcohol, other substances were reported rarely with nicotine and cannabis (each n=2) as well as specific medication-related blood pressure (n=2). Ritalin and pain killer were reported by one person each (n=1).

### Knowledge About Alcohol

Asking the question about understanding the topic of alcohol, the most frequently mentioned aspect of alcohol-related knowledge was the legal framework, specifically the age restrictions for alcohol sales in Switzerland (n=20) and the blood alcohol limit for driving (n=12). Additionally, ten interviewees (n=10) noted that no specific legal regulations exist concerning alcohol consumption for IWD. Also, there were other 12 different categories mentioned, but all of them just a few times.

#### Resources Available for Addressing Alcohol-Related Issues

When asked about resources available for addressing alcohol-related issues, many coaches were unable to name specific organizations (n=11). The most frequently mentioned resources included general counseling services such as Pro Infirmis, Dargebotene Hand (n=9), and the Blue Cross (n=8). Besides the statement mentioned only a few times, googling on the internet and contacting the regional addiction prevention center (“Regionale Suchtpräventionsstelle”) was also mentioned 8 resp. 5 times (n=8 resp. n=5).

Coaches rated their colleagues’ knowledge of available alcohol-related support services as adequate (n=13), while others assessed it as limited or nonexistent (n=7). In terms of participants’ knowledge, coaches perceived it as limited (n=12) or were unable to evaluate it (n=7).

#### Knowledge of Alcohol’s Effects on the Body and Risks

A considerable number of the surveyed coaches exhibited a limited understanding of the biological processes associated with alcohol consumption (n=13). One coach acknowledged this lack of knowledge by stating:

Honestly, I don’t really know exactly what happens or which neurotransmitters are triggered or anything.

If any knowledge was expressed, it was often in the form of superficial statements. The most commonly mentioned effects included: “alcohol enters the bloodstream” (n=5), “alcohol affects the nervous system” (n=4), “alcohol acts as a sedative” (n=2), and “alcohol is metabolized by the liver” (n=2). Several other effects were mentioned, but only by a single respondent in each case.

Regarding the risks associated with alcohol, the most frequently mentioned concerns included general health damage (n=20), with liver damage specifically cited by seven interviewees (n=7), addiction risk (n=17), and the deterioration of social relationships (n=11). Here, respondents also mentioned a variety of other possible risks solely and, in most cases, only a few times.

#### Knowledge About Interactions Between Alcohol and Medication

With respect to interactions between alcohol and medication, 12 coaches (n=12) recognized that such interactions could be dangerous, while eight (n=8) acknowledged that alcohol and medication influence each other. However, another eight coaches (n=8) admitted to having limited knowledge on this subject. The most commonly mentioned consequences of alcohol-medication interactions were unconsciousness (n=9) and reduced or absent effectiveness of the medication (n=6). Most coaches were unable to assess participants’ knowledge of these risks (n=11) or perceived it as limited (n=9).

#### Recognizing Alcohol-Related Problems

The most frequently cited indicators of alcohol-related problems included the regularity of alcohol consumption (n=17), alcohol odor (n=14), signs of dependence (n=12), and noticeable behavioral changes (n=12). One coach described dependence as follows:

[…] when it becomes a need. If it becomes a problem when having a drink is impossible.

Another coach emphasized behavioral changes as an important indicator:

If I notice every time [I see this person] that something in their behavior became different from what it used to be.

#### Strategies and Rules for Low-Risk Alcohol Consumption

The most frequently mentioned strategies for maintaining a healthy approach to alcohol consumption included monitoring the quantity of alcohol consumed (n=12) and adhering to societal norms regarding alcohol use (n=11). One coach explained:

I think healthy alcohol consumption in society, as practiced by most people, is something like having a beer in the evening or a glass of wine with dinner - that’s okay. The societal norms that exist for healthy consumption.

Another frequently mentioned strategy was avoiding drinking on an empty stomach (n=10).

#### Overall Assessment of Alcohol-Related Knowledge

Coaches rated their colleagues’ knowledge of strategies for low-risk alcohol consumption as moderate (n=9), adequate (n=7), or high (n=7). Participants’ knowledge, however, was generally rated as limited (n=9) or difficult for coaches to assess (n=9). Only two persons assumed the knowledge was high (n=2), and another four (n=4) assumed it was moderate.

In general, the surveyed coaches assessed other coaches’ knowledge about alcohol as high (n=14) or moderate (n=10). Regarding participants, they rated their knowledge about alcohol as high (n=8), moderate (n=9), or limited (n=7). However, every third coach also stated that it is challenging to make an overall statement in this case (n=9).

### Results Related to Parental Codes Questions

In order to provide a comprehensive summary of the results, we have structured our findings into four key themes inspired by the structure of the interview guide. For the present article, quotes have been translated into English.

#### Overall Situation Regarding Alcohol and Disabled Sports

Interviewees expressed heterogeneous views regarding alcohol consumption in the context of disability sports. However, their responses could be broadly categorized into three dominant perspectives: (1) alcohol and disability sports are incompatible (n=7), (2) alcohol and sports are inherently linked (n=5), and (3) alcohol consumption is not an issue in disability sports (n=4).

Beyond the direct context of sports programs, the vast majority of interviewees (n=24) acknowledged that alcohol consumption was a relevant topic outside the sporting environment. However, within this group, most respondents (n=14) considered alcohol use in such settings as unproblematic, with four interviewees (n=4) explicitly stating that alcohol consumption among participants remained below health-risk thresholds. In contrast, a minority of coaches (n=5) reported encountering participants with concerning alcohol consumption patterns. Among those who recognized alcohol use as a topic outside of sports, the most frequently cited contexts for consumption included leisure activities (n=10), social gatherings following training sessions (n=10), and club events (n=8). As one coach described:

Of course, we sometimes go out for drinks after training. And, yes, when it comes to celebrations, alcohol tends to be involved. It’s more of an issue during the social part after sports, but not otherwise.

Regarding alcohol consumption patterns, opinions on differences between coaches and participants varied. While a few interviewees suggested that coaches generally consume less alcohol than participants (n=3) or at least refrain from drinking in their presence (n=2), the most frequently held view (n=9) was that there is no notable difference in alcohol consumption patterns between the two groups. Additionally, six interviewees (n=6) indicated that coaches tended to consume more alcohol than participants.

Overall, coaches strongly advocated for equal treatment of individuals with and without disabilities concerning alcohol-related discussions. Nevertheless, one-third of respondents (n=9) emphasized the importance of assessing whether a person with a disability should consume alcohol. This concern was not attributed to disability per se but rather to the potential interaction between alcohol and prescribed medications or the possibility of simultaneous polysubstance use. One interviewee articulated this perspective as follows:

And I think caution is always necessary when you don’t know exactly whether the person is allowed to drink, whether they are on medication, and how they might react to it.

#### Assumed Reaction to Alcohol Consumption

When asked about their anticipated response to detected alcohol consumption in connection with training or its aftermath, interviewees reported a range of possible actions, as summarized in Table 1.Despite these reactions, interviewees described additional potential reactions, albeit mentioned only infrequently. These included accepting occasional consumption, contacting a club supervisor, consulting with another coach, or referring the individual to a counselling or advisory service specializing in substance use. Such responses were suggested for both coaches and participants.

**Table 1:** Assumed Reaction to Alcohol Consumption

Reaction Regarding		Reaction Towards…	
		…Another Coach	… A Participant
		n_interviewees_ (n/N %)	
Detected alcohol consumption in connection with training			
	Address the issue directly (eg, verbal intervention)	20 (74)	21 (77)
	Exclude the intoxicated individual from training	10 (37)	15 (55)
	By sending home	8 (30)	10 (37)
	By letting watch	–	5 (19)
	Terminate/suspend the individual	5 (19)	–
	Contact the responsible care giver	n/a	7 (26)
	Monitor the situation for potential recurrence	–	4 (15)
Observed excessive consumption occurred outside the disability sports program	Address the issue directly (eg, verbal intervention)	8 (30)	14 (52)
	“None of my business”	5 (19)	11 (41)

**Note**: Full sample (N=27); only responses mentioned at least four times are included.

**Abbreviation**: n/a, not applicable.

Moreover, interviewees indicated that their decision-making regarding alcohol consumption would be influenced by awareness of participants’ key medications that should not be combined with alcohol (n=17). Additionally, instructions or communication from caregivers or institutions (n=8) were frequently mentioned as factors shaping responses. Five respondents (n=5) cited general regulations on alcohol consumption in sports as a determinant of their actions.

Conversely, a substantial proportion of interviewees (n=11) emphasized the absence of official guidelines addressing alcohol consumption in disability sports. This lack of regulation was often highlighted through statements such as:

I’m not aware of any regulations. I don’t know if they exist or not.

#### Need for Action

The responses regarding whether the surveyed coaches perceive a need for action regarding alcohol consumption in disability sports were nearly evenly divided, with 11 interviewees (n=11) indicating that action is required, while 10 respondents (n=10) stated that no intervention is necessary within the disability sports programs they supervise. One coach exemplified the latter perspective by stating:

Well, in my group, I don’t see it as such a big issue. From my perspective, it’s not necessarily urgent.

In addition, seven interviewees of them expressed the opinion that, in general, no specific measures are needed to address alcohol consumption in disability sports. One coach articulated this position as follows:

In my opinion, no. Because it has to do with common sense and the sense of responsibility of the supervisors.

Among those who identified a need for action, the most frequently mentioned measures included increasing awareness of alcohol-related issues in schools or institutions (n=4) [working with the target population] and providing training programs for coaches on alcohol in disability sports (n=4). As one coach explained:

It might be good to offer a course or send materials to the coaches to bring the topic back to everyone’s attention.

Finally, four interviewees (n=4) stated they were uncertain whether action is needed, with opinions divided between pro and contra.

#### Approaches to Alcohol Prevention in Disability Sports

When asked about strategies for alcohol prevention in disability sports, the surveyed coaches described various approaches. The majority (n=15) emphasized the importance of conveying knowledge and raising awareness about alcohol-related issues. A predominant preference was for interactive and experiential learning methods, with nine interviewees (n=9) specifically advocating for playful approaches, such as role-playing exercises or alcohol simulations. One coach illustrated this perspective as follows:

I think it shouldn’t be done just through words or cognitively demanding knowledge. I think it should be done through experiences. Not by actually making people drink or anything like that, but by helping them experience what these drinks mean. It should be done playfully, like through theater or workshops.

Another frequently mentioned strategy involved direct discussions with participants about alcohol consumption (n=11). Coaches expressed a preference for facilitating open conversations in an accessible manner. As one interviewee described:

I would try to ask them about their alcohol consumption and whether they know people who drink too much alcohol, and then discuss this with them in a very simple way, weighing the pros and cons.

Additionally, several coaches emphasized the importance of providing appealing alternatives to alcoholic beverages (n=4). This approach was seen as a way to normalize non-alcoholic choices while maintaining the social and celebratory aspects often associated with drinking. One coach provided a concrete example:

Offer enjoyable non-alcoholic drinks. Let them mix their own, find recipes, decorate them nicely, and celebrate it as you would with a cocktail, wine, or beer. Celebrate it without needing alcohol.

Furthermore, several other suggestions were mentioned, though mostly by fewer than three interviewees. These included additional ideas such as providing access to counseling services, using digital tools for prevention, showing educational videos, and teaching alcohol-related knowledge or behavioral patterns based on specific situations, etc.

#### Views on Disability Sports and Alcohol Consumption From the Point of View of Inclusion

This section examines the perspectives of the surveyed coaches on the relationship between inclusion, alcohol consumption, and the autonomy of IWD.

A predominant theme among respondents was the importance of self-determination, with 17 interviewees (n=17) emphasizing that IWD should have the freedom to make their own choices regarding alcohol consumption. One coach expressed this sentiment as follows:

Our participants live self-determined lives. As long as everything in the camp remains within a normal, healthy range, I wouldn’t even address the topic of alcohol.

The role of inclusion in shaping alcohol consumption behaviors among IWD was also widely discussed. While 17 coaches (n=17) believed that inclusion could influence alcohol consumption patterns, the nature of this influence varied. The most frequently cited concern was the risk of peer pressure, mentioned by eight respondents (n=8), highlighting the potential for social environments to encourage alcohol consumption. Another four individuals (n=4) made an assumption that the self-determination and the presence and easy access to the alcoholic beverage might enhance the willingness to try it, especially in the previous context of the general prohibition of alcohol by the caregiving organization. Conversely, ten coaches (n=10) argued that inclusion has little or no noticeable impact on alcohol consumption among IWD. One interviewee articulated this viewpoint by stating:

I don’t believe, for instance, that if people are not included, they drink less. I don’t think inclusion has an impact on their alcohol consumption.

Finally, one person stated explicitly that access to alcohol should be prohibited for IWD.

#### Other Substances in Disabled Sports

Next to specific questions related to alcohol, the perception of the consumption of other substances was also assessed. Interviewees were explicitly asked about cannabis/marihuana, hard drugs, medication, tobacco/nicotine, and sugar-containing products. Table 2 shows the summarized answers regarding other substances:

**Table 2:** Reported Situation with Other Substances Than Alcohol in and Around Sports

Codes	n_interviewees_ (n/N %)				
… In the Own Sports Unit	Cannabis / Marihuana	Hard Drugs	Medication	Tobacco / Nicotine	Sugar Containing Products
It is an issue	8 (30)	1 (4)	19 (70)	23 (85)	27 (100)
It is NOT an issue	16 (59)	21 (78)	3 (11)	4 (15)	n/a
Cannot say if it is an issue	7 (26)	2 (7)	5 (19)	n/a	n/a

**Note**. Full sample (N=27).

**Abbreviation**: n/a, not addressed.

The results regarding cannabis/marihuana were strongly connected to medical purposes like medication against pain and spasticity. Regarding the medication, all statements (n=23) were related to the fact that most participants are using medication, and the coaches are mostly unaware of what kind of medication and the possible side effects of it. Here, pain medication and psychiatric medication were specifically mentioned six times each (n=6).

Tobacco / Nicotine was also mentioned in almost every interview and mainly in the same way as the following quote shows:

Yes, I notice that. After the sport, we usually walk together to the tram stop, and already on the way, there are always a few people smoking a cigarette or using electronic ones. I’ve also experienced situations where some participants go outside to smoke during the activity and then come back in.

Next to the “classic” addicting substances, sugar-containing products were mentioned by every interviewee. Most of the answers were specifically related to energy drinks or other sweet beverages. Most interviewees described this issue as:

This is a huge topic, and I feel like we’re lacking a reference point.

Additionally, possible substance-unrelated addictions (SUA) from the point of view of the coaches have also been assessed. Only five (n=5) interviewees denied that SUA is a possible issue in connection with the participants of their sports units. Half of the interviewees from the other 22 (n=22) talked about “smartphone” addiction. Other answers were very heterogeneous, mentioning only one or two times such topics as shopping addiction, gambling addiction, binge-watching of series or sports, browsing the internet, etc.

## Discussion

Research on substance-related disorders among IWD has been scarce, with limited attention given to this topic in past studies. There is currently almost no dedicated research addressing this issue in Switzerland, nor do specific prevention measures exist. However, the interplay between alcohol consumption and societal inclusion dynamics may contribute to an increased risk of developing alcohol-related disorders or misuse within this population. Given the critical role of sports in the integration and inclusion of vulnerable groups, coupled with the well-documented social aspects of sports activities - often linked to alcohol consumption and also confirmed by results of the present study - this study aims to explore the current landscape of alcohol consumption within the Swiss disabled sports system. Specifically, the study investigated the perspectives of sports coaches who oversee teams and activities for IWD, providing insights into the prevalence, patterns, and potential risks associated with alcohol use in this context.

The findings from the semi-structured interviews with coaches suggest that alcohol consumption is currently not considered a relevant issue within disability sports programs. However, the results also indicate that drinking behavior in social contexts surrounding sports is present and does not appear to differ markedly from patterns observed in regular grassroots sports, particularly in settings where socialization occurs around sporting activities.

At the same time, sure warning signs emerge concerning the interplay between sports, socialization, and alcohol consumption, affecting IWD and coaches. While alcohol consumption during sports activities is strictly rejected, its acceptance in social settings surrounding sports appears more ambiguous. Coaches predominantly discussed potential reactions to excessive consumption, highlighting a spectrum of responses ranging from intervention to inaction, but did not reflect on their own attitudes toward alcohol consumption in such situations.

Notably, coaches also did not express reflections on their own attitudes towards alcohol consumption in these settings or their role in influencing their teams beyond the immediate sporting context. Moreover, their assumptions regarding appropriate responses to excessive alcohol consumption tended to be polarized when addressing participants: some indicated they would immediately confront the issue, while others viewed it as outside their responsibility. There was no mention of intermediate responses, such as monitoring the situation over time or considering the frequency of occurrences.

Another relevant finding is that coaches spoke more frequently about excessive alcohol consumption among participants than about fellow coaches. While this could indicate a possible bias due to the assumed study’s participant-centered approach, it does not fully explain why coaches referred equally to participants and fellow coaches in discussions on sports-related contexts yet primarily emphasized excessive consumption among participants.

The results also reveal notable tendencies regarding coaches’ reactions to possible alcohol consumption during sports activities. While the majority of coaches stated they would at least acknowledge alcohol consumption verbally, irrespective of whether it involved a participant or a fellow coach, exclusion from the current training was more frequently applied to participants than to coaches. At the same time, some coaches indicated an intense way of action by suspending a fellow coach from the trainer role for alcohol consumption during training, which has never been a response observed in connection with participants. This suggests that coaches’ behavioral responses to alcohol consumption may be influenced by the individual’s role within the sports setting, with different standards applied depending on whether the person consuming alcohol is a participant or a fellow coach.

The last point appears to be supported by the fact that coaches attributed a high level of knowledge regarding both low-risk alcohol consumption strategies and general alcohol-related information to their fellow coaches rather than to participants. Notably, while every third coach expressed the opinion that participants had limited knowledge in this area, none mentioned a lack of knowledge among their coaching peers. This discrepancy is particularly relevant given that interviewees themselves primarily reported only a superficial understanding of alcohol, its effects on the body, and the risks associated with its consumption. Moreover, their knowledge regarding the interactions between alcohol and other substances, strategies for low-risk consumption, and available resources or organizations providing help and support was also limited. It may also be confirmed by the fact that every second coach reported risky alcohol consumption patterns based on their own responses to the AUDIT-C. Yet, they appeared either unaware of or unwilling to acknowledge that their drinking behavior already fell within the risky consumption range.

Surprisingly, only one-third of the coaches supported implementing specific measures addressing this issue in disability sports. At the same time, it is essential to note that the proportion of those who did not see such measures as necessary was also limited to one-third, meaning that the majority did not explicitly reject the idea. The suggested measures were, however, mostly basic, focusing primarily on raising awareness and encouraging discussion. This suggests that the overall knowledge of possible strategies for addressing substance consumption appears to be relatively low among the interviewed sample. Another important and, at the same time, a surprising finding was the apparent lack of awareness or knowledge of existing recommendations regarding substance consumption in (disabled) sports among coaches. This is particularly unexpected given that prevention programs such as “cool & clean” are an integral part of Swiss Olympic’s strategy, as well as that of affiliated umbrella organizations, including the three Disabled Sports Umbrella Organizations. Furthermore, the content of “cool and clean” is embedded in educational courses at the basic level for obtaining a coaching certification. Considering these factors, it is notable that coaches reported a lack of specific guidance on substance use prevention and did not reference this well-established and widely recognized program in Switzerland, indicating that more focus on training is needed.

Furthermore, the study indicates that alcohol is not the only substance that may be relevant for prevention measures in the context of disability sports or for IWD in general. Given that marijuana was frequently reported by interviewees in connection with its use as medication - primarily for pain management or spasticity - it presents a challenge in distinguishing its role as a substance used for hedonistic or addiction-related psychoactive purposes from its primary function as a “prescribed” therapeutic agent. However, other substances, such as sugar and tobacco, appear disproportionately prevalent within this population. The observations reported by coaches align with findings from previous studies, which have highlighted higher smoking rates among IWD compared to the general population, as well as increased consumption of sugar-containing products.^94–102^

A key finding of the study was the widespread use of medication among participants in disability sports programs. As expected, most individuals are on long-term medical treatment, which often contraindicates the consumption of alcoholic beverages. However, the study revealed a clear knowledge gap among coaches regarding the biochemical and physiological effects of alcohol consumption, particularly in relation to medication interactions - an issue known as simultaneous polysubstance use. Since coaches work with a vulnerable population, ensuring they are well-informed on these topics is crucial. A structured approach to integrating medication-related knowledge into coach training programs could help address this gap, enhancing the safety and well-being of athletes with disabilities.

## Strengths & Limitations

This study provides unique insights into alcohol consumption and substance-related behaviors in disability sports, an area that has been largely underexplored in existing research. By adopting a qualitative approach through semi-structured interviews, the study offers a nuanced understanding of coaches’ perceptions, knowledge, and attitudes toward alcohol use and its implications in disability sports settings. The integration of perspectives from various disability sports organizations, sports groups as well as language regions enhances the generalizability of findings across different sports disciplines, disabilities as well regions.

One of the key strengths of the study is its exploratory character, which allows for the identification of previously overlooked issues, such as the role of socialization in alcohol consumption, the lack of implementation of existing prevention programs, and the apparent discrepancies in alcohol-related interventions between participants and coaches. Additionally, the study highlights coaches’ limited awareness of substance-related risks, providing important directions for future prevention strategies and educational interventions.

However, several limitations must be considered when interpreting the findings. First, self-reported data may be influenced by social desirability bias, particularly when discussing alcohol-related behaviors. Coaches may have even underreported their own alcohol consumption or overstated their low level of knowledge regarding prevention strategies. Second, while the qualitative approach allows for an in-depth exploration of perceptions, the sample size remains relatively small, limiting the extent to which results can be generalized to all disability sports settings in Switzerland. Furthermore, the study primarily captures coaches’ perspectives, meaning that the viewpoints of athletes with disabilities, medical professionals, and sports administrators remain underrepresented in the present paper.

Future research should aim to triangulate data collection methods, incorporating quantitative assessments and direct observational studies to validate self-reported behaviors. Additionally, expanding the study to include athletes’ perspectives and an international comparative approach could provide a more comprehensive understanding of alcohol and substance use in disability sports across different cultural and structural contexts.

## Conclusion

The findings of this study underscore the complex interplay between sports participation, socialization, and alcohol consumption in disability sports. While alcohol use is largely rejected during training and competition, its presence in social contexts surrounding sports activities is normalized, mirroring trends observed in mainstream sports. However, the study also reveals gaps in knowledge and inconsistent intervention strategies among coaches, particularly regarding alcohol’s interaction with medication, long-term health implications, and available prevention measures.

The study further identifies critical discrepancies in how alcohol consumption is addressed depending on whether the individual is a participant or a fellow coach, raising concerns about implicit biases in enforcement and policy implementation. Additionally, despite the existence of the Swiss Olympics’s “cool & clean” prevention program, coaches displayed limited awareness of its recommendations, suggesting a need for more effective dissemination and integration of existing prevention efforts.

Given the high prevalence of medication use among athletes with disabilities, the study highlights the urgent need for structured education on substance-related risks in disability sports. Providing comprehensive training for coaches on substance use prevention, harm reduction, and risk assessment should be a key priority for disability sports organizations.

Ultimately, these findings emphasize the need for targeted prevention strategies that take into account the specific vulnerabilities of athletes with disabilities. By integrating evidence-based prevention programs, fostering open discussions, and ensuring that coaches are equipped with the necessary knowledge and tools, disability sports organizations can contribute to creating a safer and more inclusive sports environment. Future research should focus on longitudinal studies assessing the impact of prevention measures and cross-sector collaborations to strengthen substance use policies in disability sports.

## Data Availability

The interview guidelines in German and French are available upon reasonable request from the corresponding author. According to the informed consent, we promised the respondents that the interview transcripts would be stored according to ISGF’s data protection requirements and would not be published/uploaded anywhere for open access. This was important because we interviewed a very sensitive group. We had to accept this obligation to ensure the interviewees spoke freely.

## Abbreviations

AUDIT-C, Alcohol Use Disorders Identification Test; BFS, Bundesamt für Statistik (Swiss Federal Statistical Office); CRPD, Convention on the Rights of Persons with Disabilities; EDI, Eidgenössisches Departement des Innern (Federal Department of Home Affairs, Switzerland); EKSN, Eidgenössische Kommission für Fragen zu Sucht und Prävention nichtübertragbarer Krankheiten (Swiss Federal Commission on Addiction and Prevention of Non-Communicable Diseases); IWD, Individuals with Disabilities; ISAMB, Inklusion im Sport: Alkoholprävention mit Menschen mit Behinderungen (Inclusion in Sports: Alcohol Prevention with Individuals with Disabilities); ISGF, Schweizer Institut für Sucht- und Gesundheitsforschung (Swiss Research Institute for Public Health and Addiction); NVivo, Qualitative data analysis software; SUA, Substance-Unrelated Addictions; WHO, World Health Organization.
